# Claiming Positive Results From Negative Trials: A Cause for Concern in Randomized Controlled Trial Research

**DOI:** 10.2196/jmir.2845

**Published:** 2013-08-19

**Authors:** John A Cunningham

**Affiliations:** ^1^Centre for Mental Health ResearchThe Australian National UniversityCanberraAustralia; ^2^Centre for Addiction and Mental HealthToronto, ONCanada

**Keywords:** research methods, randomised controlled trials, alcohol, Internet, brief intervention

One of the challenging issues facing the randomized controlled trial (RCT) researcher is how to interpret the results of studies where there are improvements in the behaviour under study but where the degree of improvement does not differ between the experimental conditions [1]. This is especially a challenge when the RCT involves the comparison of two or more interventions rather than an intervention compared to some form of no-intervention control group.

One possible cause of the observed improvement in such trials is that both interventions were “active” - that both interventions were effective in facilitating or causing a change among participants.  Unfortunately, there is no way to determine if this claim is definitely true from the results of a negative RCT. Other interpretations of the results include: 1) that the change over time is due to regression to the mean [2, 3]; 2) due to natural history maturation (meaning that participants were in a period in their lives where, on average, a downward trend in quantity of drinking could be expected); or 3) the trial recruited participants who were already motivated to change and who would have done so anyway without exposure to the interventions under study [4].

Any of these alternate explanations could apply to the recent trial by Hester and colleagues [1]. Further, there is a well-established finding in the alcohol research field that participants in the no intervention control condition of intervention trials show improvements in their drinking from baseline to follow-up [5]. This may be particularly the case in trials recruiting participants from the community rather than from treatment settings where intractable alcohol problems are more common [6]. Essentially, the assumption that any changes over time are due to the intervention in a negative trial is predicated on the assumption that the participants would show no improvement without receiving some type of intervention. There may be some behaviours where this is the case. However, alcohol abuse is demonstrably not one of them. Thus, it is unwise to favour an intervention effect explanation over other causes when faced with the results of an RCT where participants show improvement over time but that there are no significant statistical differences between intervention conditions.
